# Development and Psychometric Properties of The Delayed Childbearing Questionnaire (DCBQ-55)

**DOI:** 10.3390/healthcare6040120

**Published:** 2018-09-23

**Authors:** Samira Behboudi-Gandevani, Saeideh Ziaei, Anoshirvan Kazemnejad, Farideh Khalajabadi Farahani, Mojtaba Vaismoradi

**Affiliations:** 1 Department of Midwifery & Reproductive Health, Medical Sciences Faculty, Tarbiat Modares University, 14115-111 Tehran, Iran; behboudi@endocrine.ac.ir; 2 Department of Biostatistics, Medical Sciences Faculty, Tarbiat Modares University, 14115-111 Tehran, Iran; kazem_an@modares.ac.ir; 3 National Population Studies & Comprehensive Management Institute, 1531635711 Tehran, Iran; farideh.farahani@psri.ac.ir; 4 Faculty of Nursing and Health Sciences, Nord University, 8049 Bodø, Norway; mojtaba.vaismoradi@nord.no

**Keywords:** delayed childbearing, fertility, postponement, psychometric properties, questionnaire, reproductive health, women health

## Abstract

The comprehensive assessment of delayed childbearing needs a valid and reliable instrument. Therefore, the aim of the present study was to develop an instrument to evaluate factors influencing delayed childbearing among women and to assess its psychometric properties. The current methodological study was performed in two phases of (i) qualitative instrument development, and (ii) quantitative psychometric assessment of the developed instrument. Face and content validity of the instrument was assessed by eligible women and a panel of experts. Construct validity was assessed using the exploratory factor analysis (EFA). For reliability, internal consistency reliability and intra-rater reliability analysis were used. The initial instrument developed from the qualitative phase consisted of 60 items, which were reduced to 55 items after the face and content validity processes. EFA (*n* = 300) using the Kaiser criteria (Eigenvalues > 1) and the scree plot led to a six-factor solution accounting for 61.24% of the observed variance. The Cronbach’s alpha coefficient, Spearman’s correlation, test–retest and intra-class correlation coefficients for the whole instrument were reported as 0.83, 0.86 and 0.81, respectively. The final instrument entitled the delayed childbearing questionnaire (DCBQ-55) included 50 items with six domains of ‘readiness for childbearing’, ‘stability in the partner relationship’, ‘awareness about the adverse outcomes of pregnancy in advanced maternal age’, ‘attitude toward delayed childbearing’, ‘family support’, and ‘social support’ on a five-point Likert scale. The DCBQ-55 as a simple, valid and reliable instrument can assess factors influencing delayed childbearing. It can be used by reproductive healthcare providers and policy makers to understand factors influencing delayed childbearing and devise appropriate strategies.

## 1. Introduction

Delayed childbearing as ‘a personal choice to postpone childbearing in women over 35 years’ has become a health concern in both developed and developing countries [1,2]. It is believed that the age of a woman at the first pregnancy and the number of pregnancies in women aged over 35 years are rising across the globe [2,3]. An extensive use of family planning programs, increased popularity of assisted reproductive technology (ART), occupation and education, lack of support by the society for childbearing, lack of knowledge of the negative consequences of advanced maternal age, socioeconomic uncertainties and irresponsible partners are some of the known factors underlying delayed childbearing [4,5,6,7]. However, the postponement of childbearing can lead to a wide range of adverse social, health, and demographic outcomes for the mother and child. For instance, it carries the risk of infertility, obstetric complications, pregnancy-associated chronic diseases and neonatal health issues [8,9,10]. 

Delayed childbearing is a significant risk factor for low birth weight, but some positive associations have been reported between the maternal profile and birth outcomes among women aged ≥ 35 years [11]. Nevertheless, it can create some challenges to maternity care in terms of the spacing of a woman’s pregnancies. It is recommended to create a balance between inter-pregnancy intervals associated with higher risks for adverse pregnancy outcomes and increased maternal age at delivery [12]. In terms of psychological issues, those women who are childless after delaying childbearing experience similar feelings to those women that are childless after infertility [13]. Therefore, couples’ understandings of the planning and timing of parenthood, as well as the impact of female and male age on the ability to achieve parenthood, should be improved [14]. 

Delayed childbearing is an evolving global issue across the world with a wide range of clinical and social outcomes. For instance, a study in the USA on the basis of national birth data showed an increasingly prominent trend of delayed childbearing from 1971 to 2016. Only in 2016, delayed childbearing accounted for 24% and 38% of multiple births for white and black women, respectively. It was predicted that by 2025, delayed childbearing would account for higher rates of multiple births [15]. Postponement of childbearing can have a tremendous effect on the total fertility rate [16,17]. In the EU-28 Member States, the total fertility rate has steadily declined from the mid-1960s, but since the 2000s, some signs of increase have been reported. In 2010, a subsequent reduction was observed, followed by a slight increase towards 2016 [18]. With regard to the Iranian fertility context, the introduction of family planning services in the 1960s and increased marriage age during the 1970s reduced the fertility rate. Socioeconomic factors including gender roles, changes in women’s education and workforce participation have mainly influenced the trend of long-term fertility in Iran [19]. Since 1988, the development of the economy and living standards has led to changes in the population policy in terms of fertility control programs [20]. Fertility in Iran has been reported to be constant from 2000 to 2009 at a level of 1.8–2.0 births per woman, indicating an effort to sustain fertility at the replacement level. However, a population bill in 2013 showed that the fertility rate in Iran had fallen to a low level, and a wide range of policies were devised to increase it to 2.5 births per woman [21]. The report by the Iran’s national statistics organization in the period of 2012–2016 showed that the total fertility rate was 2.01 children [22]. In a recent cross-sectional study in 2015 in Tehran on 1067 married women participating in the Tehran Lipid and Glucose Study (TLGS), the overall prevalence of lifetime primary infertility was reported as 17.3%, which was higher than its global trend [23]. Iranian women are largely unaware of the potential complications of delayed childbirth and its relationship with infertility [24]. It is believed that a short postponement of motherhood by Iranian women has occurred since 1990 onwards. An Iranian study (2013) showed that childlessness between ages 15–39 increased during 1991–2003, but was reduced from 3.8% to 2.2% in the last years of reproductive age. In addition, voluntary and involuntary childlessness among married women were reported as 8.5% and 2.0%, respectively [25]. The recent report by Iran’s national statistics organization (2017) showed the highest increase in fertility was observed within the age group of 35–39 in urban areas. It was mentioned that such a change in the age pattern greatly influenced the fertility rate to the replacement level in Iran [22]. 

Knowledge of factors influencing delayed childbearing is important for the improvement of maternal and child care. However, there is a lack of appropriate and specific instrument for the assessment of delayed childbearing, both in the Iranian society and across the globe. A valid and reliable instrument for the assessment of delayed childbearing can cover multidimensional aspects of this phenomenon and comprehensively explore factors influencing it, given cultural and regional differences in various contexts [26]. Therefore, the aim of the present study was to develop an instrument to evaluate factors influencing delayed childbearing among women and to assess its psychometric properties.

## 2. Material and Methods

Devising the instrument for a comprehensive assessment of various dimensions of delayed childbearing needed a context-based study and a thorough literature search. Therefore, this methodologic study was consisted of the two phases of (i) qualitative (to define the components of delayed childbearing and develop the initial instrument) and (ii) quantitative (to assess the psychometric properties of the instrument) [27]. 

### 2.1. The Qualitative Phase

The details of this phase have been described elsewhere [2]. Briefly, subjects were 23 nulliparous married women, aged ≥ 30 years, who voluntarily postponed childbearing. They were selected using the purposive sampling method on the basis of following inclusion criteria: decided to postpone childbirth for at least five years, attended three prenatal or gynecology healthcare clinics in Tehran for various health-related reasons, and were willing to participate in this study [2]. They were informed of the aim and process of the study, and signed the written informed consent form before data collection. Next, 28 sessions of in-depth semi-structured interviews, lasting 20–40 min, were held in places convenient to them. Each woman was requested by the first author (SBG) to describe her own individual perspective and experience about all aspects of delayed childbearing with a focus on the following questions: ‘What is your understanding of delayed childbearing? Why have you decided to delay childbearing? What factors influenced your decision to delay childbearing? Are you satisfied with your decision?’ Also, branching questions were asked to follow their thoughts and increase the interviews’ depth. A conventional content analysis approach [28,29] was used for data analysis, concurrently with data collection. The interviews were discontinued when no new data was collected and data saturation was reached. Main themes developed in this study were “personal inclination”, “perceived beliefs about delayed childbearing”, and “social support” [2]. 

In addition, the researchers conducted a thorough review of literature in Farsi and English to retrieve published articles and instruments on delayed childbearing. International academic databases such as PubMed (including Medline), Scopus, Science Direct, Cinahl, Web of Science and Iranian databases that provided the highest yield of citations on the study topic up to 2015 were searched. Furthermore, for maximizing coverage, a manual search of the reference lists of related articles were performed. The search key words included ‘childbearing’, ‘delay’, ‘postponement’, ‘fertility’, and ‘childlessness’. The search yielded 2356 potentially relevant articles. The titles and abstracts of the initial list of articles were reviewed by the researchers independently and 21 articles were selected on the basis of the following inclusion criteria: focused on factors influencing delayed childbearing, published in peer-reviewed journals, and being available online. The contents of the selected articles were used for item generation. No specific instrument for the assessment of factors influencing delayed childbearing was found in the literature.

### 2.2. The Quantitative Phase

In the second phase, the psychometric properties of the preliminary instrument were evaluated. Validity of the instrument was established through the assessment and confirmation of content, face and construct (exploratory factor analysis (EFA)) validities. For reliability, internal consistency and test–retest analyses were performed. Details of this phase have been described as follow:

#### 2.2.1. Initial Item Generation

An initial 60-item instrument was developed based on the extracted themes and related codes from the qualitative study and the thorough literature search [30]. It used a five-point Likert scale (1 = strongly disagree to 5 = strongly agree) for respondents to show their level of agreement/disagreement with each item.

#### 2.2.2. Face Validity

Face validity was performed to investigate the women’s understandings of the instrument items. Qualitative and quantitative methods of face validity were used. For qualitative evaluation of face validity, 10 women with delayed childbearing were requested to assess each item in terms of ‘difficulty’, ‘irrelevance’, and ‘ambiguity’. Also, they were asked to provide feedbacks and give additional suggestions for the improvement of the initial instrument and rectify potential mistakes. As such, the quantitative assessment of face validity was performed using the item impact score reflecting the subjects’ perceptions of the items’ importance on a five-point Likert scale. The item impact was defined as the proportion of the women who identified it as ‘important’ and the mean importance score attributed to the item (impact score = frequency * importance). The satisfactory score for the acceptance of each item was ≥ 1.5 [30].

#### 2.2.3. Content Validity

Content validity helped determine whether the items adequately addressed the construct of delayed childbearing. An expert panel consisting of 15 multidisciplinary specialists in the fields of midwifery, reproductive health, obstetrics and gynecology, maternal–child health, nursing, community health, psychology, and sociology evaluated the content validity of the initial instrument using the Waltz and Bausell content validity index (CVI) [30]. They scored the ‘relevancy’, ‘clarity’, and ‘simplicity’ of each item using a four-point Likert scale, and the CVI for each item was calculated by dividing the number of specialists who scored items three or four by the total number of specialists. The item was accepted if the CVI was ≥ 0.79 [30]. The necessity of the items was assessed using a three-point rating scale as (i) not necessary, (ii) useful, but not essential, and (iii) essential. Following the experts’ evaluation, a content validity ratio (CVR) for the total scale was also calculated. According to the Lawshe table, an acceptable CVR value for the 15-expert panel was reported as 0.49 [30].

#### 2.2.4. Construct Validity

A cross-sectional study was performed to assess the construct validity of the initial instrument using the EFA with the principal components method and varimax rotation. The recommended number of subjects for the EFA was recommended as five times the number of items [30,31]. Therefore, 300 women aged ≥ 35 who had experienced a time in their life when they decided to postpone childbearing for at least five years on the basis of personal reasons and attended three healthcare centers were selected. Those women who suffered from chronic serious illnesses which substantially affected their ability to experience pregnancy, such as high-grade heart failure or linguistic or cognitive problems, and also those who did not provide the answer to at least 20% of all items were excluded from data analysis. 

The characteristics of the women were presented in Table 1. After explaining the aim of the study and obtaining signed written informed consent, they were requested to provide responses to the instrument’s items. The Kaiser-Meyer-Olkin (KMO) test was used to evaluate the sample adequacy [31], and the cut-off point of 0.40 was considered the minimum load factor required for maintaining each item of the factor being extracted [31].

#### 2.2.5. Reliability

Reliability of the instrument was examined using internal consistency and test–retest (test of stability across time) analyses. The Cronbach’s alpha coefficients were calculated for subscales and the whole instrument to evaluate the internal consistency for which a value of ≥0.6 was accepted for descriptive studies [32]. Stability of the instrument was examined using the test–retest analysis conducted on 25 women with delayed childbearing who completed the questionnaire twice within a two-week interval [33]. The intra-class correlation coefficient (ICC) was also calculated and classified as follows: 0.0–0.2 as low, 0.21–0.40 as fair, 0.41–0.60 as moderate, 0.61–0.80 as substantial, and 0.81–1 as almost perfect [34]. 

#### 2.2.6. Statistical Analysis

The SPSS software for Windows version 16.0 was used to perform all statistical analyses (SPSS Inc., Chicago, IL, USA, 2008). Both item- and subscale-level analyses were conducted using descriptive statistics including frequencies, means and standard deviations. The statistical analysis of construct validity was performed through the EFA with the principal component method and varimax rotation. Eigenvalues of more than one and a scree plot were used to determine the number of factors. Factor loadings equal or greater than 0.4 were considered appropriate. The Cronbach’s alpha coefficient and ICC were also calculated. *p*-values < 0.05 were set as statistically significant.

### 2.3. Ethical Considerations

The Research and Ethics Committee affiliated with Tarbiat Modares University (decree code: 15-6-2014) approved the study research protocol. The women were informed of their rights and the possibility of withdrawal from the study at any time. They were also ensured that the data collection was confidential and would be used only for the research purpose. Also, the written informed consent form was signed by all women who willingly agreed to take part in this study. 

## 3. Results

The total number of initial items generated during the qualitative phase and literature review were 82 items. After eliminating redundancies by the research team, they were reduced to 60 items. The classification of these 60 items under the three themes resulting from qualitative content analysis was as follows: personal inclination (18 items), perceived beliefs about delayed childbearing (15 items), and social support (27 items).

During face validity, some typographical errors were corrected and also all items achieved the minimum impact score required for inclusion. In content validity, the number of items was reduced from 60 to 57, since the CVR was less than 0.49. The mean CVR in this study for the total scale was reported as 0.87, indicating a satisfactory result. As such, two items did not achieve the CVI above 0.79 and were omitted from the final questionnaire. The mean CVR and CVI for the scale were 0.87 and 0.92, respectively, indicating appropriate content validity. 

For the identification of the underlying factor structure of the instrument, the EFA was conducted using a principal components analysis. A total of 300 women with the experience of delayed childbearing (mean age of 37.8 years) agreed to complete the 55-item questionnaire entitled The Delayed Childbearing Questionnaire (DCBQ-55).

**Figure 1:**
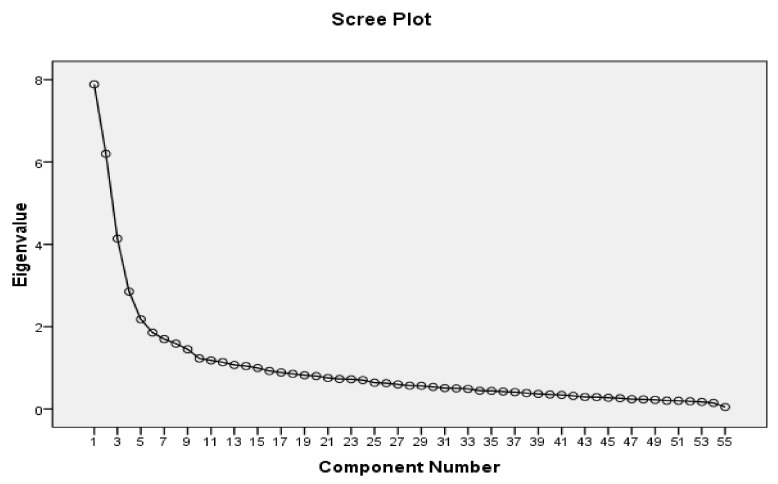
Scree plot for The Delayed Childbearing Questionnaire (DCBQ-55) (*n* = 300).

The KMO coefficient was reported as 0.809 (*p* < 0.001), indicating that the properties of the correlation matrix justified conducting the EFA. A varimax rotation identified six latent factors. The extraction was based on the visual interpretation of the scree plot (Figure 1) and Kaiser’s criterion for Eigenvalues ≥ 1. The six factors jointly accounted for 61.24% of the observed variance. No item was deleted due to adequate loading on the factors. However, due to its further compatibility, one item from domain five was transferred to domain six. Table 2 provides the details, factors, labels and the number of items. 

The Cronbach’s alpha coefficient was reported as 0.836. The ICC was reported as 0.81 indicating a suitable stability of the instrument. Table 3 provides the description of the Cronbach’s α coefficient and ICC for the instrument and its domains. For stability through the test–retest analysis, Spearman’s correlation coefficient was reported as 0.86.

## 4. Discussion

In this methodological study, both qualitative and quantitative approaches were used to design an applicable instrument for the assessment of factors influencing delayed childbearing. In addition, it was shown that the DCBQ-55 met psychometric requirements including reliability, validity, and internal consistency for use in community and clinical settings. The main characteristic of the DCBQ-55 is to directly address the perceptions and concerns of women regarding delayed childbearing. It includes 55 items in six domains of ‘readiness for childbearing’, ‘stability in the partner relationship’, ‘awareness about the adverse outcomes of pregnancy in advanced maternal age’, ‘attitude toward delayed childbearing’, ‘family support’, and ‘social support’, enabling researchers and policymakers to assess the various aspects of this phenomenon overlooked in previous studies. To our knowledge, prior to the DCBQ-55, there was no specific instruments for the assessment of factors influencing delayed childbearing. The use of qualitative and quantitative approaches and a thorough literature search have led to the development of an instrument that can help with a systematic assessment of this phenomenon. For instance, in terms of reliability, the acceptable level of internal consistency of the DCBQ-55 was reported. It meant that each item in this instrument was highly correlated with the total score, suggesting that the items were homogeneous and measured a similar overall assessment’s construct.

The first domain of the DCBQ-55 was ‘readiness for childbearing’. According to the international literature, lack of readiness by couples has been be linked to their awareness of the sacrifices and costs of parenthood, and the common notion that parenthood can be delayed safely. Couples need to improve their knowledge of the age-related decline in fertility and its impact on future parenthood [14]. Creating a positive atmosphere regarding childbearing can influence a couple’s decision on the postponement of childbearing [35].

‘Stability in the partner relationship’ was another domain of the instrument developed in this study. Koert and Daniluk reported that women were delaying childbearing to seek an appropriate partner [13]. For many women, enduring marital relationships is associated with better health-related decisions and outcomes in life [36]. On the other hand, making a decision on the family size is adjusted and explained by changes in the partner. If there is a decision of childbearing postponement until the age of 30 years, it is more likely to lower the family size than if the childbearing career is started earlier [37].

With regard to ‘awareness about the adverse outcomes of pregnancy in advanced maternal age’, as the third dimension of the DCBQ-55, a matched case-control study in women aged over 35 years in comparison with younger mothers in Nigeria showed that delayed childbearing after 35 years was often not associated with adverse pregnancy outcomes. In other words, it could indicate women’s awareness of the potential risks of pregnancy and increased use of obstetric services [38]. According to another study in Denmark, the increased risk of infertility, spontaneous abortions, ectopic pregnancies and trisomy 21 started at around 30 years of age, and the increasing risk of preterm births and stillbirths started at around 35 years of age. Therefore, increasing couples’ awareness of the probable negative effects of advanced maternal age on reproductive outcomes is crucial [39].

‘Attitude toward delayed childbearing’ was the fourth dimension of this instrument. A recent study in Canada showed that women ≥ 35 years of age believed themselves to have more knowledge regarding age-related pregnancy risks than those under the age of 35 years. However, they preferred to receive further counselling about it. This study also demonstrated that women’s attitudes did not correlate with their measured knowledge. Therefore, continuous and face-to-face education regarding the age-related risks of pregnancy is required to improve their health literacy [40]. It is also believed that individuals’ values may directly impact behaviors related to reproductive health [41]; however, the effect of personal values and being informed and encouraged about the timing of pregnancy during young adulthood needs further longitudinal evaluations [42].

The fifth dimension of the DCBQ-55 was ‘family support’. It has been stated that women’s perceptions of family support may have a negative association with family planning unmet needs among women [43]. Therefore, support systems should be devised with the aim of improving women’s access to maternity services, especially culturally appropriate services that encompass community, stakeholder options and respect for their cultural preferences [44].

The last dimension of the DCBQ-55 was ‘social support’. It is noted that maternity-related decisions are mainly associated with conflicts and difficulties as a result of family values, religious beliefs and presence of social and healthcare support. Social and educational institutions need to become more pregnancy-friendly to encourage women to have an early pregnancy [45]. In addition, women will benefit from increased support in the workplace and from insurance systems for fertility preservation and healthy reproduction [41].

The DCBQ-55 includes the sexual-related issues of delayed childbearing that have been overlooked by previous studies. While this section is highly cultural [46], it provides important information for a more precise assessment of delayed childbearing among women. In this respect, the high prevalence of sexual-related problems among pregnant women [47], the common notion that sexual relationships are forbidden during pregnancy [48,49], prolonged sexual abstinence after childbirth for ensuring family health, and the social implications of non-adherence to sexual abstinence norms [50] can negatively impact couples’ emotional and sexual life [2].

Since no appropriate and culture-contextual instrument was available to assess delayed childbearing, concurrent validity could not be examined in this study. Nevertheless, the comprehensive literature review performed in the first phase of instrument development enriched the item pool and ensured that the DCBQ-55 could be used by researchers in other cultures and contexts. Also, the study’s subjects in both the qualitative and quantitative phases were female, but childbearing decision-making might be mostly a couple’s decision. Therefore, future studies need to involve males in data collection to provide a more comprehensive picture of delayed childbearing. In this study, only women that postponed childbearing based on their own personal choices and in controlled situations were recruited. However, some other women may perceive that they have no ultimate control on the timing of childbearing [51]. Future studies need to consider the perspectives of such women, men and healthcare providers involved in making a decision on delayed childbearing to further revise the DCBQ-55 and improve its generalizability.

## 5. Conclusions

Delayed childbearing is an important contributor to women’s maternity experiences and requires greater attention in clinical and policy-making decisions related to the family planning process. The DCBQ-55 as a simple, valid and reliable instrument opens the way for a more comprehensive assessment of factors influencing delayed childbearing. The average time to complete the DCBQ-55 by a participant is about 10–15 min, indicating that the DCBQ-55 is quick to complete and easy to score. The researchers suggest the incorporation of culture-contextual aspects of delayed childbearing during data collection using the DCBQ-55 and interpretation of findings. The DCBQ-55 can feed information to reproductive healthcare providers and policy makers to make appropriate decisions regarding delayed childbearing in line with population development policies.

## Figures and Tables

**Table 1:** Characteristics of the subjects in this study.

Variable	Response (*n* = 300)
Age, mean (SD), y	36.1 (8.2)
Marital age, mean (SD), y	8.2 (3.9)
Husband’s age, mean (SD), y	40.1 (5.7)
Education level, *n* (%)	
≥High school graduate	162 (54)
Bachelor’s degree	90 (30)
Postgraduate	48 (16)
Job status, *n* (%)	
Employed	192 (64)
Unemployed	108 (36)
Household income *, *n* (%)	
Poor	54 (18)
Middle	216 (72)
Well-off	30 (10)

* Self-reported.

**Table 2:** Factors, items and factor loadings of The Delayed Childbearing Questionnaire (DCBQ-55)* (*n* = 300).

Items	Factor 1	Factor 2	Factor 3	Factor 4	Factor 5	Factor 6
Factor 1: Readiness for childbearing						
Being sure about physical health before pregnancy	0.821	0.471	0.017	0.067	0.074	0.017
Control of chronic diseases before pregnancy	0.819	0.215	0.063	0.084	0.051	0.036
Having mental peace before pregnancy	0.815	0.015	0.074	0.128	0.121	0.097
Ability to transfer mental safety and security to the spouse before pregnancy	0.798	0.122	0.005	0.014	0.224	0.215
Having concerns about being a good mother and wife at the same time	0.777	0.168	0.084	0.157	0.168	0.254
Meeting the basic necessities before pregnancy	0.763	0.101	0.054	0.108	0.185	0.135
Having suitable financial savings before pregnancy	0.755	0.020	0.035	0.114	0.117	0.136
Having a suitable and secure job before pregnancy	0.745	0.187	0.150	0.168	0.231	0.198
Ability to provide the best facilities for childcare	0.732	0.096	0.220	.0164	0.226	0.146
Completing education and studies before pregnancy	0.703	0.124	0.221	0.064	0.117	0.318
Having concerns about financial problems during pregnancy	0.620	0.183	0.012	0.134	0.091	0.018
Having concerns about losing better economic situations during pregnancy	0.581	0.163	0.187	0.078	0.107	0.015
Lack of responsibility and commitment to have a child	0.506	0.043	0.024	0.093	0.208	0.147
Lack of self-confidence to be a mother	0.506	0.123	0.014	0.215	0.018	0.095
High costs of pregnancy and childbirth	0.319	0.106	0.054	0.197	0.064	0.031
Factor 2: Stability in the partner relationship						
Achieving a comprehensive understanding of the spouse before pregnancy	0.33	0.860	0.214	0.057	0.163	0.069
Being sure of the reliability of the spouse for the rest of life	0.051	0.741	0.011	0.001	0.002	0.101
Developing strong relationships with the spouse	0.43	0.714	0.197	0.036	0.102	0.111
Feeling concerns about the ability to have enjoyable sexual relationships during pregnancy	0.33	0.707	0.72	0.128	0.114	0.022
Feeling concerns about personal attractiveness during pregnancy	0.87	0.686	0.233	0.043	0.210	0.185
Necessity of having children to achieve peace and stability in life	0.051	0.317	0.114	0.045	0.084	0.036
Factor 3: Awareness about the adverse outcomes of pregnancy in advanced maternal age						
Increased risk of infertility in women older than 35 years	0.047	0.124	0.617	0.165	0.036	0.182
Increased risk of obstetrics complications in advanced maternal age	0.088	0.065	0.611	0.109	0.062	0.126
Increased risk of neonatal complications in advanced maternal age	0.047	0.125	0.524	0.068	0.085	0.051
Presence of effective treatments for infertility to solve related problems	0.091	0.054	0.454	1.25	0.150	0.055
Losing the chance of having a second child due to delayed childbearing	0.158	0.026	0.409	0.001	1.05	0.201
Factor 4: Attitudes toward delayed childbearing						
Possibility of having a child at any age without facing any problem if God willing	0.103	0.156	0.048	0.667	0.114	0.165
Completion of the woman’s identity through motherhood	0.147	0.121	0.039	0.633	0.065	0.195
Giving a meaning to the life through having a child	0.031	0.114	0.094	0.591	0.069	0.168
Importance of bringing a male or female child	0.085	0.032	0.129	0.588	0.142	0.024
Delayed childbearing as an interference in God’s affairs	0.044	0.052	0.152	0.580	0.063	0.065
Creating a balance in the family decision-making between man and woman by delayed childbearing	0.163	0.098	0.231	0.572	0.117	0.096
Reduction of the women’s power in the family due to early childbearing	0.047	0.187	0.011	0.570	0.016	0.048
Sufficiency of having only one child for the family	0.214	0.025	0.053	0.545	0.021	0.057
Starting a family later in life due to marriage at the old age	0.001	0.064	0.121	0.454	0.133	0.045
Factor 5: Family support						
Being under pressure by the couple’s families for delayed childbearing	0.062	0.147	0.036	0.080	0.656	0.037
Getting help from the couple’s families for taking care of the child	0.142	0.015	0.199	0.142	0.614	0.091
Successful experiences of delayed childbearing in the couple’s families	0.070	0.088	0.078	0.058	0.602	0.158
Persuasion of the couple to delay childbearing due to being raised in a small paternal family	0.018	0.011	0.148	0.046	0.581	0.036
Factor 6: Social support						
Childbearing as one of the most important social functions of the family	0.091	0.044	0.015	0.190	0.247	0.701
Being encouraged by others to delay childbearing	0.035	0.088	0.107	0.047	0.675	0.692
Limitations in the freedom for getting engaged in social activities due to childbearing	0.125	0.121	0.044	0.013	0.214	0.671
Delayed childbearing as a sign of modernity	0.017	0.014	0.125	0.166	0.118	0.657
Popularity of early childbearing in families with a lower social status	0.094	0.055	0.087	0.122	0.147	0.649
Education regarding delayed childbearing in schools and universities	0.225	0.024	0.096	0.085	0.129	0.629
Free access to modern contraceptive methods	0.019	0.035	0.067	0.101	0.155	0.625
Lack of childcare facilities at the workplace	0.195	0.128	0.088	0.032	0.138	0.624
Giving the responsibility of child care to a nursery with confidence	0.109	0.165	0.036	0.080	0.091	0.590
The high cost of childcare in a nursery	0.068	0.135	0.166	0.063	0.050	0.581
The short period of maternity leave for employed mothers	0.36	0.100	0.109	0.085	0.075	0.570
Support of delayed childbearing in mass media	0.015	0.121	0.094	0.021	0.150	0.564
Lack of special laws to support pregnant women or mothers	0.190	0.134	0.096	0.048	0.021	0.562
Being fired from work due to getting pregnant	0.087	0.087	0.148	0.046	0.107	0.513
Threating the child due to social insecurity	0.046	0.050	0.018	0.080	0.075	0.507
Lack of a bright future to start a family	0.011	0.010	0.115	0.198	0.114	0.481

* The permission to use the DCBQ-55 in future studies is granted by the authors ONLY with a full citation to this article.

**Table 3:** Descriptive statistics and reliability measurements of the DCBQ-50. CI = confidence interval.

Domain	Item (Number)	Cronbach’s Alpha	ICC (95% CI)	Spearman’s Correlation Coefficient (*n* = 25)	*p*-Value
Readiness for childbearing	15	0.833	0.83 (0.81–0.87)	0.71	0.001
Stability in the partner relationship	6	0.735	0.69 (0.67–0.74)	0.91	0.001
Awareness about the adverse outcomes of pregnancy in advanced maternal age	5	0.844	0.83 (0.80–0.88)	0.85	0.001
Attitude toward delayed childbearing	9	0.839	0.82 (0.79–0.89)	0.96	0.001
Family support	4	0.830	0.83 (0.79–0.85)	0.91	0.001
Social support	16	0.815	0.70 (0.68–0.84)	0.86	0.001
Total	55	0.836	0.81 (0.79–0.86)	0.86	0.001
